# Supplementary material for: Metagenomic analysis of sewage for surveillance of bacterial pathogens: A release experiment to determine sensitivity
Source: PLoS One. 2024 May 16;19(5):e0300733. doi: 10.1371/journal.pone.0300733 (PMC11098379; doi:10.1371/journal.pone.0300733)
Supplement: S1 File — (DOCX) [file pone.0300733.s001.docx]

**Supplementary tables**

| Time interval | Sample name | Flow rate  [m^3^/min] | Sewage per interval [m^3^] |
| --- | --- | --- | --- |
| 10:00-11:00 | K07_1A | 6.00 | 360 |
|  | K07_1B |  |  |
| 11:01-11:30 | K07_2A | 6.38 | 185 |
|  | K07_2B |  |  |
| 11:31-12:00 | K07_3A | 5.17 | 150 |
|  | K07_3B |  |  |
| 12:01-12:30 | K07_4A | 5.45 | 158 |
|  | K07_4B |  |  |
| 12:31-13:00 | K07_5A | 5.41 | 157 |
|  | K07_5B |  |  |
| 13:01-14:00 | K07_6A | 4.93 | 291 |
|  | K07_6B |  |  |
| 14:01-15:00 | K07_7A | 5.00 | 295 |
|  | K07_7B |  |  |
| 15:01-16:00 | K07_8A | 5.32 | 314 |
|  | K07_8B |  |  |
| 16:01-17:00 | K07_9A | 5.03 | 297 |
|  | K07_9B |  |  |
| 17:01-18:00 | K07_10A | 4.81 | 284 |
|  | K07_10B |  |  |

Table S1: Sampling intervals and flow rates for release day 1. The suffices A and B in the sample names refer to subsample 1 and 2, respectively.

| Time interval | Sample name | Flow rate  [m^3^/min] | Sewage per interval [m^3^] |
| --- | --- | --- | --- |
| 10:00-11:00 | K08_1A | 7.40 | 444 |
|  | K08_1B |  |  |
| 11:01-11:30 | K08_2A | 7.21 | 209 |
|  | K08_2B |  |  |
| 11:31-12:00 | K08_3A | 6.55 | 190 |
|  | K08_3B |  |  |
| 12:01-12:30 | K08_4A | 6.97 | 173 |
|  | K08_4B |  |  |
| 12:31-13:00 | K08_5A | 6.38 | 185 |
|  | K08_5B |  |  |
| 13:01-14:00 | K08_6A | 5.61 | 331 |
|  | K08_6B |  |  |
| 14:01-15:00 | K08_7A | 5.78 | 341 |
|  | K08_7B |  |  |
| 15:01-16:00 | K08_8A | 5.22 | 308 |
|  | K08_8B |  |  |
| 16:01-17:00 | K08_9A | 5.36 | 316 |
|  | K08_9B |  |  |
| 17:01.18:00 | K08_10A | 5.32 | 314 |
|  | K08_10B |  |  |

Table S2: Sampling intervals and flow rates for release day 2. The suffices A and B in the sample names refer to subsample 1 and 2, respectively.

| Time interval | Sample name | *S hyicus*  fragmentCount | Sample  fragmentCount |
| --- | --- | --- | --- |
| 10:00-11:00 | K07_1A | 2 | 21891595 |
|  | K07_1B | 0 | 31041211 |
| 11:01-11:30 | K07_2A | 0 | 44567919 |
|  | K07_2B | 1 | 20925585 |
| 11:31-12:00 | K07_3A | 0 | 31652301 |
|  | K07_3B | 0 | 24272489 |
| 12:01-12:30 | K07_4A | 0 | 36773125 |
|  | K07_4B | 0 | 27229829 |
| 12:31-13:00 | K07_5A | 341 | 27262720 |
|  | K07_5B | 1306 | 32375124 |
| 13:01-14:00 | K07_6A | 1784 | 23789122 |
|  | K07_6B | 2126 | 25615464 |
| 14:01-15:00 | K07_7A | 1424 | 23721081 |
|  | K07_7B | 2344 | 32137050 |
| 15:01-16:00 | K07_8A | 284 | 21910265 |
|  | K07_8B | 338 | 31742210 |
| 16:01-17:00 | K07_9A | 26 | 14984708 |
|  | K07_9B | 30 | 25288426 |
| 17:01-18:00 | K07_10A | 48 | 22896227 |
|  | K07_10B | 38 | 25899363 |

Table S3. Release and mapping results on release day 1. Columns are time interval for sampling of sewage, name of sample, fragment counts for *Staphylococcus hyicus* and total fragment count for the sample, respectively. The suffices A and B in the sample names refer to the splitting up original sewage material into two independently treated subsamples.

| Time interval | Sample name | *S hyicus*  Fragment count | Sample  Fragment count |
| --- | --- | --- | --- |
| 10:00-11:00 | K08_1A | 0 | 17389732 |
|  | K08_1B | 0 | 25112958 |
| 11:01-11:30 | K08_2A | 0 | 14518301 |
|  | K08_2B | 0 | 28763663 |
| 11:31-12:00 | K08_3A | 0 | 19261771 |
|  | K08_3B | 0 | 19491276 |
| 12:01-12:30 | K08_4A | 545 | 28407073 |
|  | K08_4B | 581 | 27258506 |
| 12:31-13:00 | K08_5A | 8052 | 17807610 |
|  | K08_5B | 12625 | 22256210 |
| 13:01-14:00 | K08_6A | 4486 | 23728307 |
|  | K08_6B | 4370 | 17153297 |
| 14:01-15:00 | K08_7A | 1172 | 27452856 |
|  | K08_7B | 834 | 22170771 |
| 15:01-16:00 | K08_8A | 213 | 22247603 |
|  | K08_8B | 205 | 32574558 |
| 16:01-17:00 | K08_9A | 25 | 31822941 |
|  | K08_9B | 46 | 30412585 |
| 17:01-18:00 | K08_10A | 1 | 27111598 |
|  | K08_10B | 6 | 21686619 |

Table S4: Release and mapping results on release day 2. Columns are time interval for sampling of sewage, name of sample, fragment counts for *Staphylococcus hyicus* and total fragment count for the sample, respectively. The suffices A and B in the sample names refer to the splitting up original sewage material into two independently treated subsamples.
