# Supplementary material for: Metagenomic analysis of sewage for surveillance of bacterial pathogens: A release experiment to determine sensitivity
Source: PLoS One. 2024 May 16;19(5):e0300733. doi: 10.1371/journal.pone.0300733 (PMC11098379; doi:10.1371/journal.pone.0300733)
Supplement: S2 File — (PDF) [file pone.0300733.s002.pdf]

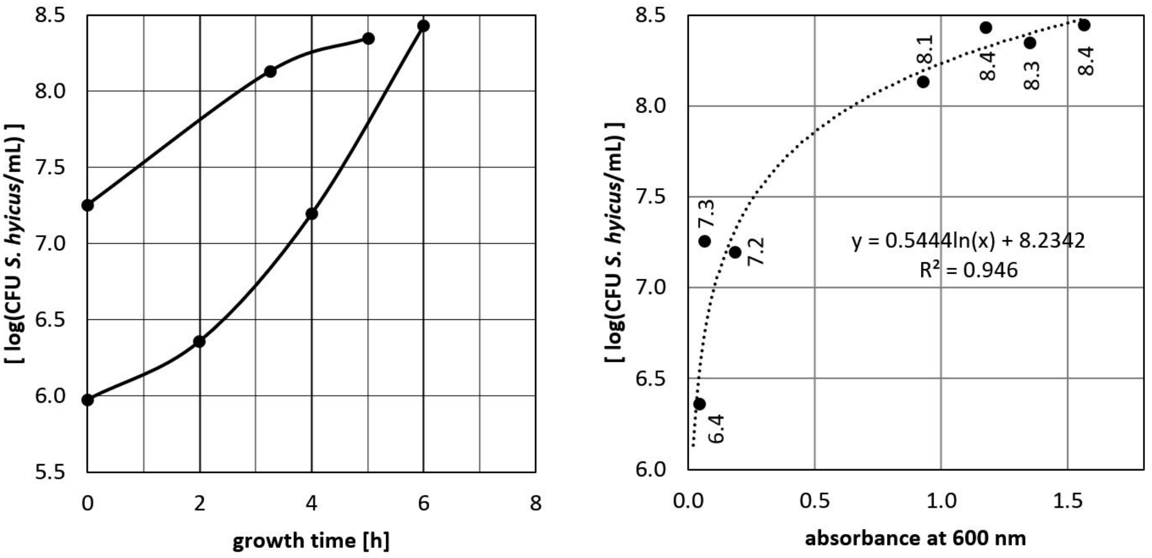

Figure S1 (left): Growth curve for *S. hyicus* over time at high inoculation rate (1:100) to assess exponential growth time from 10^7 to >10^8 CFU. Upper curve with preheated medium, lower cource with medium at 5° C and initial lag phase. (right): Photo spectrometry-cell titre correlation for *S. hyicus* with trend line, corresponding formula and regression value.

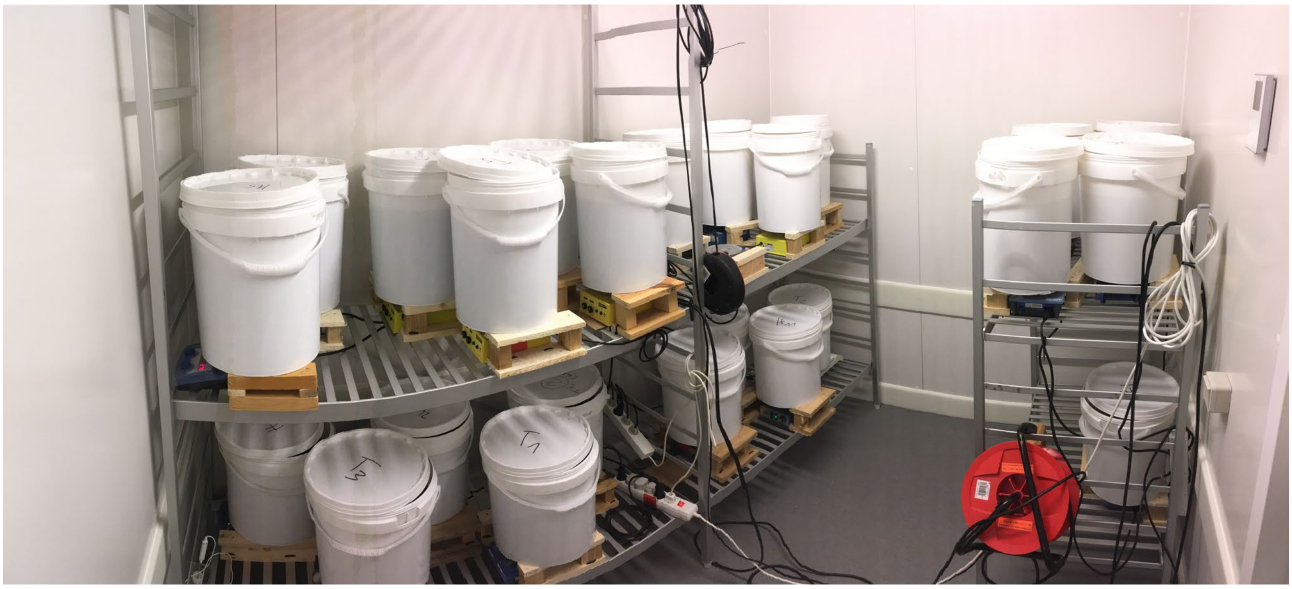

Figure S2: Setup for the final enrichment of 2×182 L in 24 22-L buckets. Walk-in incubator with wooden jigs to secure incubation vessels over magnetic stirrers.

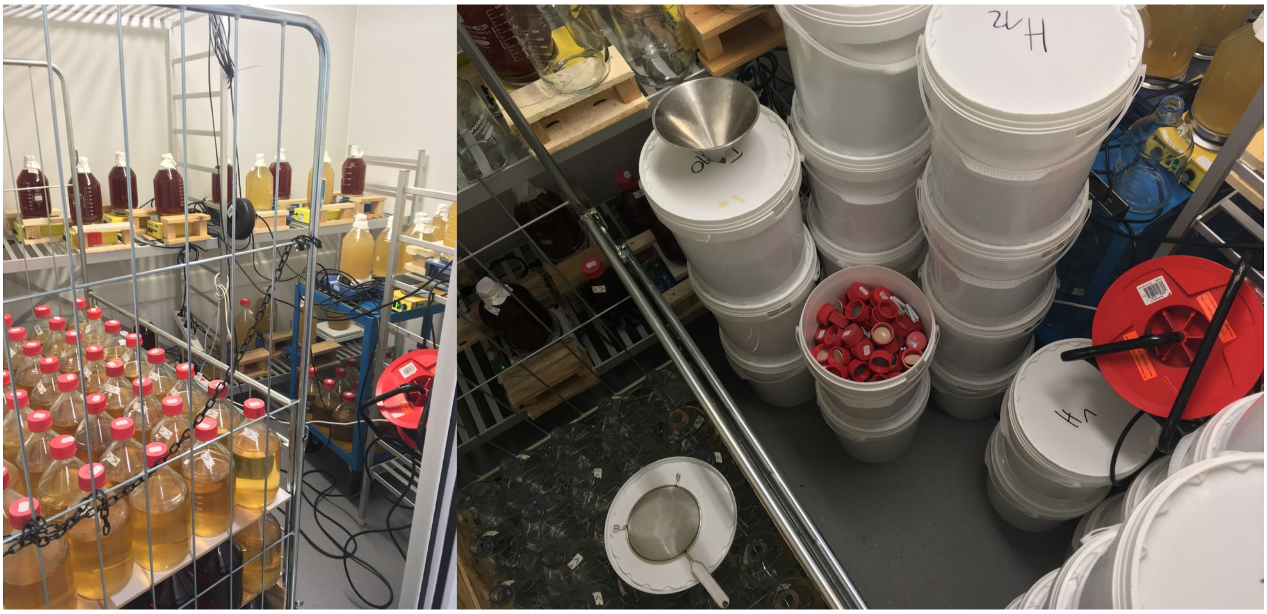

Figure S3 (left): Walk-in incubator with the third enrichment step in progress in Schott flasks covered with white filter paper. In the trolley on the left is the media for the fourth enrichment, pre-heating in Schott flasks with red caps on. (right): Pooling of the third enrichment in a covered fashion inside the walk-in incubator; a funnel inserted through a hole in lid and a sieve to catch the stirring magnets. All around, piles of 22-L buckets containing the media for the fourth enrichment.

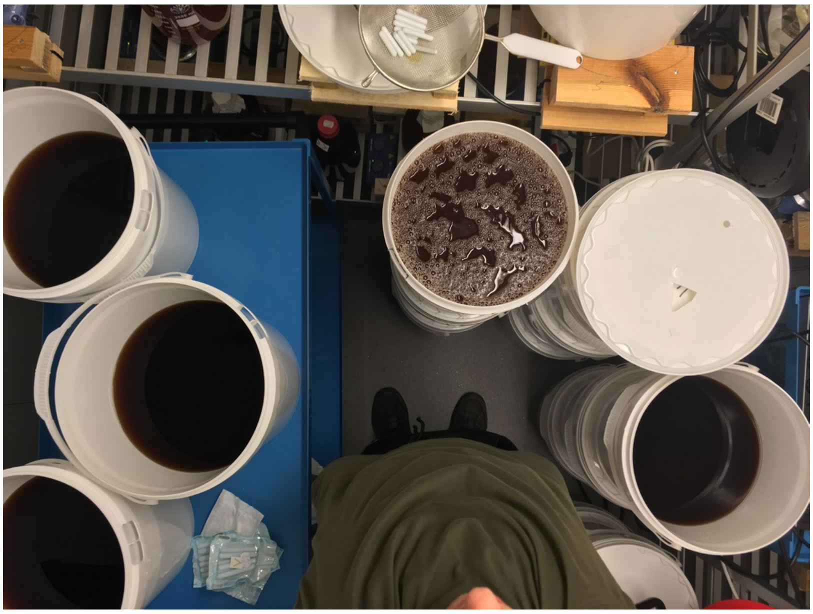

Figure S4: Final inoculation in progress by means of a 1-L ladle. Pooled, third enrichment in the middle. Aliquots of media in white buckets on either side, ready for inoculation. New, sterile stirring magnets ready in the left-hand bottom corner. An inoculated bucket already sat on its stirrer in the right-hand top corner.

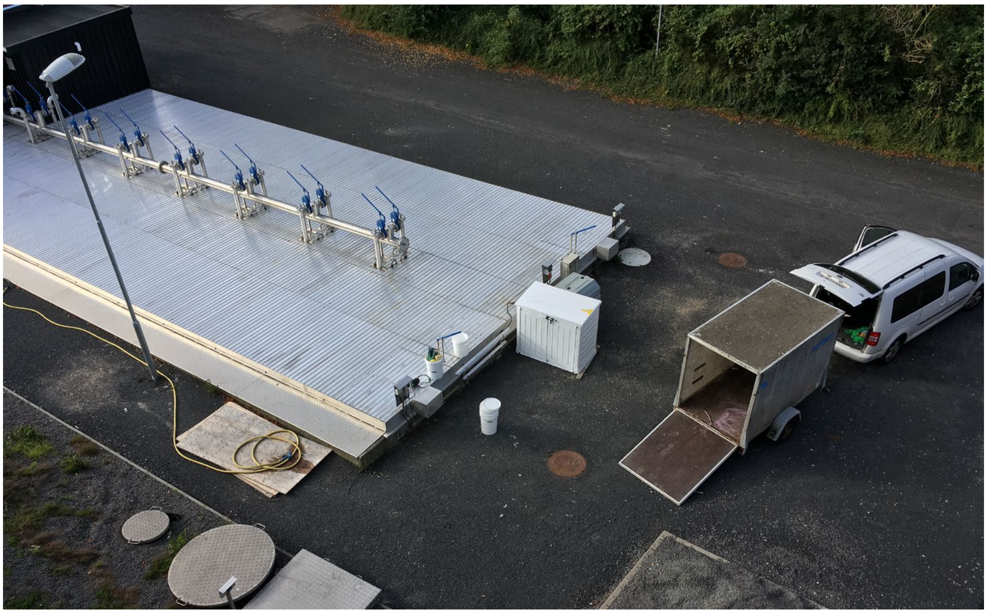

Figure S5: White, continuous sampling station at the end of sedimentation basin for grit, facing away from the weather side. Behind, the WWTP's own discontinuous sampling station (grey) and flow meter.

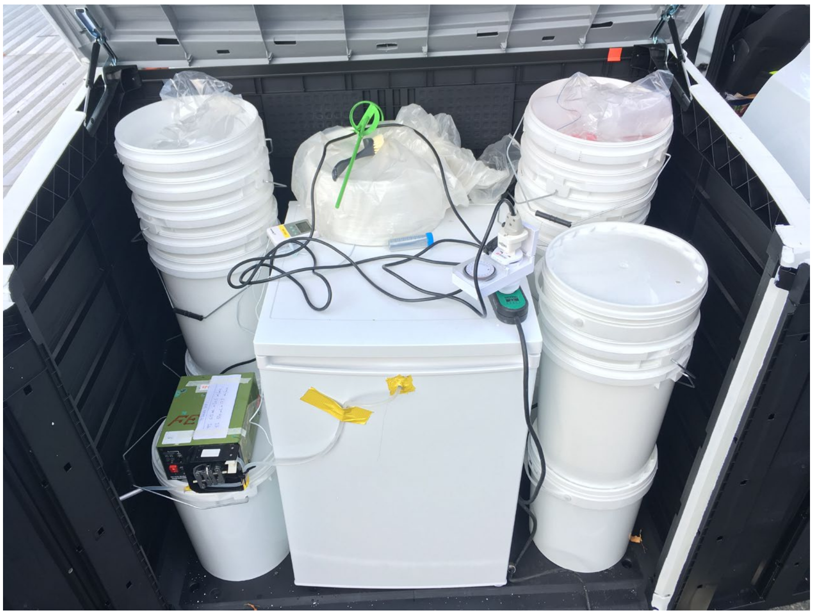

Figure S6: View onto the open sampling station, weighed-down in the corners by four 22-L buckets, filled with water. The white sampling tube enters in the left-hand bottom corner, runs through the peristaltic pump, enters the front of the fridge and the bucket inside through a hole in the lid.

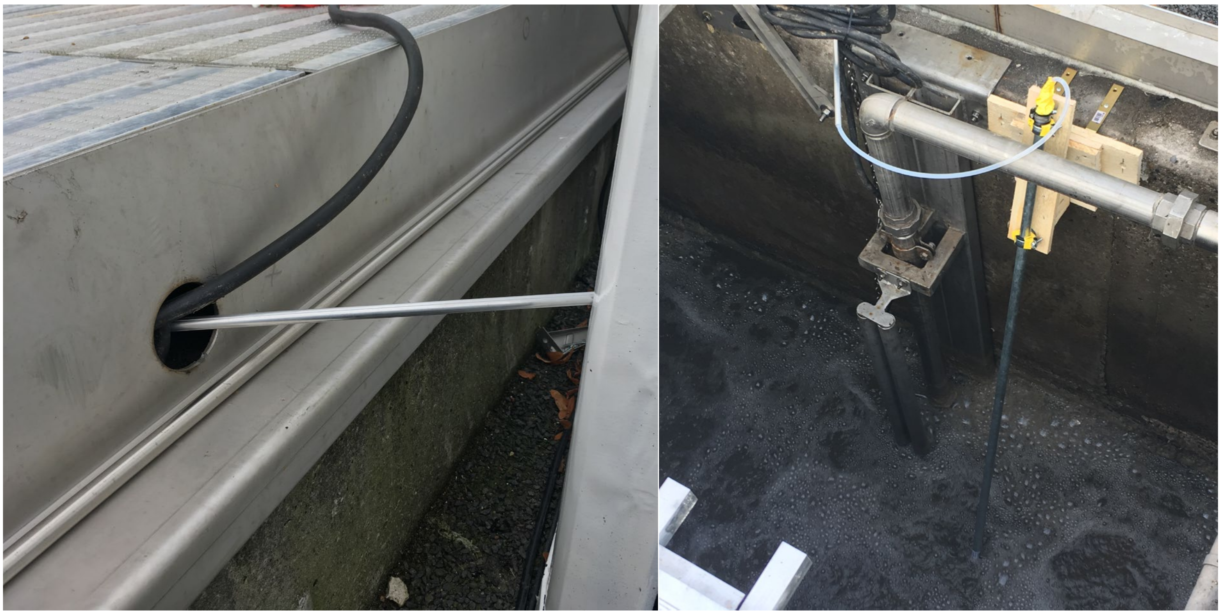

Figure S7 (left): The sampling box on the right and the face of the housing of the sedimentation basin on the left. A metal tube connects the two and holds the peristaltic tube to provide mechanical and UV protection. (right): The open end of the sedimentation basin. The peristaltic tube enters the metal pipe, suspended into the sewage at a 21° angle, and the submerged sampling head.

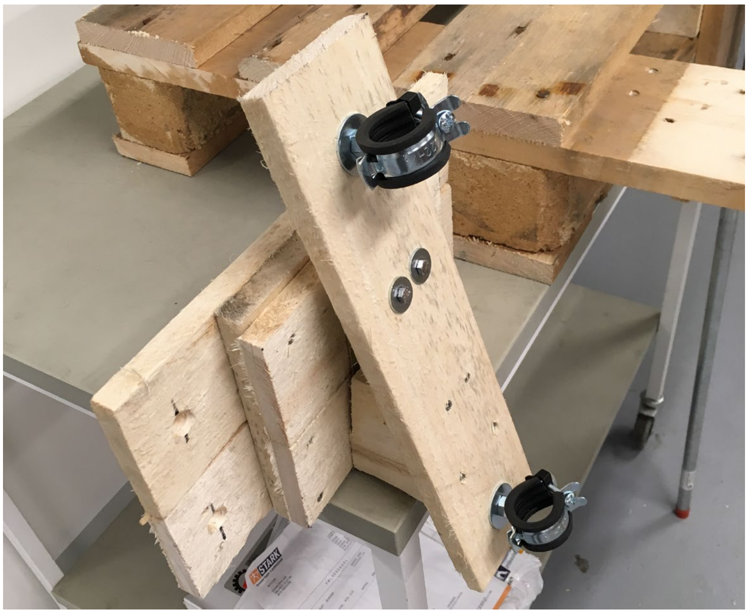

Figure S8: Jig from scrap wood to mount the sampling lance against the inside of the sedimentation basin. Metal braces with rubber grummets hold the pipe in place and prevent it from sliding.

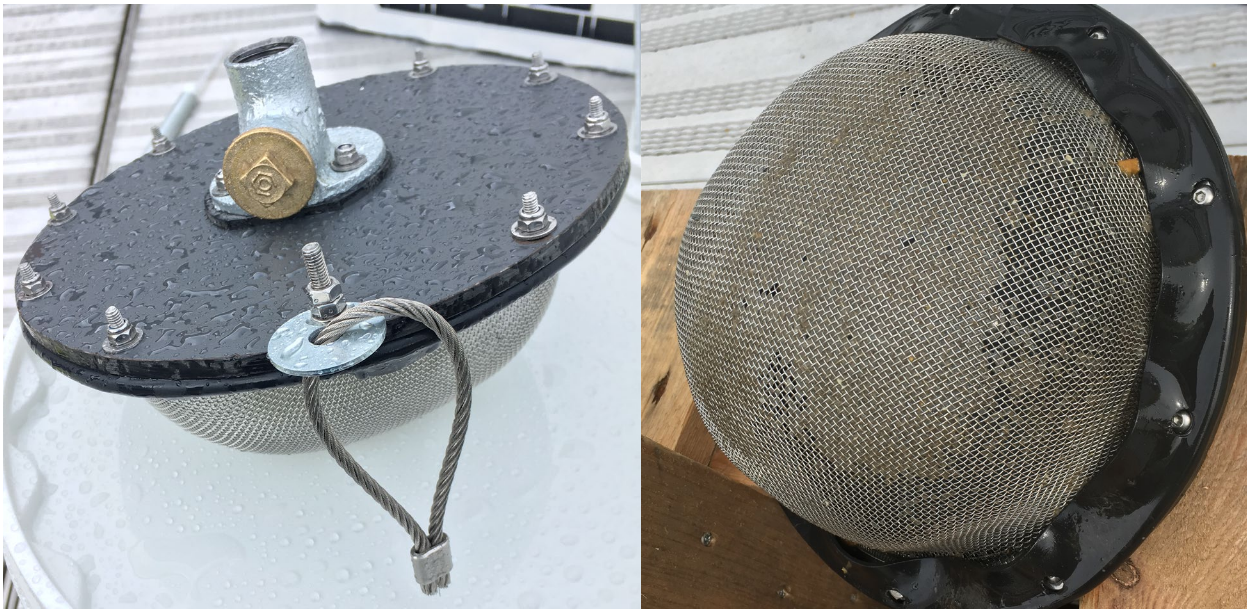

Figure S9: Side view of the sampling head. A fine mesh kitchen appliance sieve, mounted against a plastic back plate with a U-shaped rubber seal around the sieve's edge. A piece of connective plumbing hardware, mounted onto the back on a rubber seal, screws onto the metal pipe. A hole drilled through the centre of the connective piece and back board allows the peristaltic tube to protrude half-way into the sieve. (right): The mesh of the sampling head bears only minor signs of clogging after three-days' exposure to the inlet stream.
