# Supplementary material for: Metagenomic analysis of sewage for surveillance of bacterial pathogens: A release experiment to determine sensitivity
Source: PLoS One. 2024 May 16;19(5):e0300733. doi: 10.1371/journal.pone.0300733 (PMC11098379; doi:10.1371/journal.pone.0300733)
Supplement: Attachment — Submitted filename: Response to reviewers.docx [file pone.0300733.s003.docx]

PONE-D-23-32545

Metagenomic Analysis of Sewage for Surveillance of Bacterial Pathogens: A Release Experiment to Determine Sensitivity

PLOS ONE

**Comments from the editor:**

5. We note that the grant information you provided in the ‘Funding Information’ and ‘Financial Disclosure’ sections do not match.

When you resubmit, please ensure that you provide the correct grant numbers for the awards you received for your study in the ‘Funding Information’ section.

6. Thank you for stating the following financial disclosure: "Main funder is the European Union"
Please state what role the funders took in the study.  If the funders had no role, please state: ""The funders had no role in study design, data collection and analysis, decision to publish, or preparation of the manuscript."" 
If this statement is not correct you must amend it as needed. 
Please include this amended Role of Funder statement in your cover letter; we will change the online submission form on your behalf.

7. Thank you for stating the following in the Acknowledgments Section of your manuscript: "This study was supported by the European Union’s Horizon 2020 research and innovation programme under grant agreement no. 874735 (VEO) and The Novo Nordisk Foundation (NNF16OC0021856: Global Surveillance of Antimicrobial Resistance).
We note that you have provided funding information that is not currently declared in your Funding Statement. However, funding information should not appear in the Acknowledgments section or other areas of your manuscript. We will only publish funding information present in the Funding Statement section of the online submission form. 
Please remove any funding-related text from the manuscript and let us know how you would like to update your Funding Statement. Currently, your Funding Statement reads as follows: "Main funder is the European Union"

Has been included in the cover letter

Please include your amended statements within your cover letter; we will change the online submission form on your behalf.

8. Please include captions for your Supporting Information files at the end of your manuscript, and update any in-text citations to match accordingly. Please see our Supporting Information guidelines for more information: <http://journals.plos.org/plosone/s/supporting-information>.

Has been included

9. We note that Figure S5 in your submission contain [map/satellite] images which may be copyrighted. All PLOS content is published under the Creative Commons Attribution License (CC BY 4.0), which means that the manuscript, images, and Supporting Information files will be freely available online, and any third party is permitted to access, download, copy, distribute, and use these materials in any way, even commercially, with proper attribution. For these reasons, we cannot publish previously copyrighted maps or satellite images created using proprietary data, such as Google software (Google Maps, Street View, and Earth). For more information, see our copyright guidelines: <http://journals.plos.org/plosone/s/licenses-and-copyright>.
 
We require you to either (1) present written permission from the copyright holder to publish these figures specifically under the CC BY 4.0 license, or (2) remove the figures from your submission:

I have deleted the figure and changed the numbering accordingly

**Reviewer comments**

[Note: HTML markup is below. Please do not edit.]

Reviewers' comments:

Reviewer's Responses to Questions

**Comments to the Author**

1. Is the manuscript technically sound, and do the data support the conclusions?

The manuscript must describe a technically sound piece of scientific research with data that supports the conclusions. Experiments must have been conducted rigorously, with appropriate controls, replication, and sample sizes. The conclusions must be drawn appropriately based on the data presented.

Reviewer #1: Yes

Reviewer #2: Yes

2. Has the statistical analysis been performed appropriately and rigorously?

Reviewer #1: Yes

Reviewer #2: Yes

3. Have the authors made all data underlying the findings in their manuscript fully available?

The [PLOS Data policy](http://www.plosone.org/static/policies.action#sharing) requires authors to make all data underlying the findings described in their manuscript fully available without restriction, with rare exception (please refer to the Data Availability Statement in the manuscript PDF file). The data should be provided as part of the manuscript or its supporting information, or deposited to a public repository. For example, in addition to summary statistics, the data points behind means, medians and variance measures should be available. If there are restrictions on publicly sharing data—e.g. participant privacy or use of data from a third party—those must be specified.

Reviewer #1: Yes

Reviewer #2: Yes

4. Is the manuscript presented in an intelligible fashion and written in standard English?

PLOS ONE does not copyedit accepted manuscripts, so the language in submitted articles must be clear, correct, and unambiguous. Any typographical or grammatical errors should be corrected at revision, so please note any specific errors here.

Reviewer #1: Yes

Reviewer #2: Yes

5. Review Comments to the Author

Please use the space provided to explain your answers to the questions above. You may also include additional comments for the author, including concerns about dual publication, research ethics, or publication ethics. (Please upload your review as an attachment if it exceeds 20,000 characters)

Reviewer #1: The manuscript aims to investigate the sensitivity of metagenomic sequencing for the detection of specific sewage bacteria in the context of wastewater-based epidemiology. This was conducted in situ by spiking known quantities of extraneous bacteria (Staphyloccus hyicus) into a sewer system before metagenomic sequencing of samples from the associated wastewater treatment plant. The authors highlight the potential for wastewater metagenomics to estimate the number of sick individuals with an increased sensitivity over previous non-metagenomic methods.

Overall, I found the manuscript to be well written, interesting, and novel as relatively few published studies have performed spiking/spot-release experiments in the context of wastewater-based epidemiology. However, I do believe clarification of some points, potential further analyses and additional discussion would enhance the manuscript.

I have following specific comments.

1. Line 137-140: Since these initial values are pivotal to the overall experiment and calculations of sensitivity, please provide more detailed justification on why a 200 g feces assumption was made (supporting reference?) and the same for CFU per gram - is based on observed amounts from Campylobacter spp. and Salmonella ssp infection stool samples? If so, please provide supporting reference.

THERE IS AS FAR AS WE CAN TELL A SURPRISINGLY LIMITED NUMBER OF STUDIES THAT HAVE QUANTIFIED AMOUNT OF FECES EXCRETED BY HEALTH AND DIARRHEAL PEOPLE AND ALSO VERY LIMITED STUDIES QUANTIFYING THE AMOUNT OF PATHOGENS. WE HAVE INCLUDED SOME REFERENCES, BUT THIS CERTAINLY WARRENTS FURTHER STUDIES.

2. Line 220-222: The metagenomic sequencing depth is not clearly reported but can be partially inferred through the reported total fragments in Tables 1 and 2 (Line 299-311) or from the discussion (Line 328). This is of crucial importance and relevance to the manuscript analyses since sensitivity to detect specific targets in metagenomes is directly tied to metagenomic sequencing depth and sample complexity (<https://doi.org/10.1186/s40793-019-0347-1>). Please report the sequencing depth achieved per sample and discuss the impact of sequencing depth as a limitation (i.e. deeper sequencing would likely be required due to reduced/scattered real world release as compared to the large experimental spot-release)

Added average sequencing depth in results section and the individual sample depths are in S3 and S4.

3. Line 294-317: This section is very interesting and highlights potential sensitivity differences between results based on multiple samples and a single peak sample (as seen in other studies), but this is not currently mentioned in the discussion. Was there any factor which could explain the reduced sensitivity in Day 1-peak conc (e.g. weather, flow, taxonomic composition apart from S. hyicus). It would also be interesting to compare the sensitivities for all single samples and not just the sample with peak S. hyicus concentration

We have looked at the data trying to explain the increased sensitivity on day 2 which was 3 and 4 times higher, using either all fragment count data (table 2) or only fragment counts from the single sample with maximum S. hyicus (table 3). It seems that the observed abundance of S. hyicus in the samples is a major factor i.e. P(A). We did not do it for all single samples except for the one with maximum amount of observed S. hyicus. The explanation is elaborated in Discussion section.

4. Line 320-343: The discussion section is very limited in scope and does not address several presented results which warrant discussion such as the similarities and differences between sensitivity of compound sample results (Table 1) and peak conc. (Table 2) – as above.

Much alike the above explanation we also tried to explain the sensitivity difference in terms of P(B) which also include the amount of released S. hyicus in terms of the approximated ‘number of sick people N’. Using P(B) was slightly better at explaining the sensitivity differences compared to the P(A) factor. This is also elaborated in Discussion section.

5. Line 329: The calculation of the value “1 in 260 to 2,250 people” is not clearly described. It can be inferred that it originates from from Tables 1 and 2 using Minimum amount required (n) and normalised by size of the WWTP through the conclusion only (Line 349-351). Please clarify at first mention.

This has been clarified in the discussion where we show the formula used in the calculations.

Minor comments/typographical issues:

Line 32: Shotgun is more commonly accepted over “shot-gun” – please update

This is now corrected

Line 139: Please update citation format to be consistent with numbering used throughout
We have now included the reference

Line 226: Please reference where the sequences were downloaded from to generate the reference database “genomic2_20191017”
This is now explained in the methods section under ‘*DNA-sequencing and metagenomic analyses’*

Line 230: Please provides reference for Spades as done for KMA (Line 225-226)

For Frank: added full reference in the end of manuscript and just wrote (Nurk et al. 2017) in text but that needs to be formatted correctly

Line 336: Typo “Cation” should be Caution

Now corrected

Reviewer #2: The manuscript, entitled "Metagenomic Analysis of Sewage for Surveillance of Bacterial Pathogens: A Release Experiment to Determine Sensitivity", provided new information about the implication of metagenomics for detecting pathogens from sewage. The manuscript is well-written and organized.

THANK YOU

The results can be applied to monitoring bacterial pathogens.

THANK YOU

For the methods, could you please explain the reason for using only the CFU of 1.66 and 1.91 but not different lower concentrations?
IT WAS PURELY A QUESTION OF RESSOURCES. WE AGREE THAT ADDITIONAL EXPERIMENTS SHOULD BE DONE, BUT THAT WILL NOT CHANGE THE MAIN POINT, NAMELY THAT THE SENSITIVITY CAN BE LOW.

The second question is about the real sample from the community. The experiment revealed the sensitivity of the method for tracing the pathogens. However, is the method good for the real situation when the community has the patients caused by other pathogens? It would be more effective if the authors applied this method to a specific pathogen.

AGREED. HOWEVER, RELEASING FORT EXAMPLE A PATHOGENIC SALMONELLA BACTERIUM WOULD COME WITH BOTH ETHICAL AND TECHNICAL CONCERNS. RELEASING A KNOWN PATHOGEN COULD BE CONSIDERED A HUMAN HEALTH RISK AND FURTHERMORE, ANY SIGNALS FROM THE RELEASED ORGANISM COULD BE MIXED WITH NATURALLY OCCURING INFECTIONS.

IN THIS CASE WE DECIDED ON A PROVEN NON-PATHOGENIC ANIMAL ASSOCIATED BACTERIAL SPECIES TO LIMIT HUMAN HEALTH RISKS AND ALSO A BACTERIAL THAT HAVE NOT BEEN DETECTED IN ANY SEWAGE WE HAVE PREVIOUSLY ANALYSED.

Thank you.
